# 25-Hydroxyvitamin D and Severity of Parkinson's Disease

**DOI:** 10.1155/2013/689149

**Published:** 2013-07-17

**Authors:** Ahmad Chitsaz, Mohammad Maracy, Keivan Basiri, Maryam Izadi Boroujeni, Amir Pouya Tanhaei, Marzieh Rahimi, Rokhsareh Meamar

**Affiliations:** ^1^Isfahan Neurosciences Research Center, Isfahan University of Medical Sciences, 8174675731 Isfahan, Iran; ^2^Department of Neurology, Medical School, Isfahan University of Medical Sciences, 8174675731 Isfahan, Iran; ^3^Department of Biostatistics & Epidemiology, School of Public Health, Isfahan University of Medical Sciences, 81745 Isfahan, Iran; ^4^Department of Medical science, Islamic Azad University, Najafabad Branch, 517 Isfahan, Iran

## Abstract

*Introduction*. A role for vitamin D deficiency in Parkinson's disease (PD) has recently been proposed. Given the growing body of evidence for the association of vitamin D with several neurodegenerative disorders and unavailability of any published study in the Middle East, the present study is aimed to determine the associations of circulating 25-hydroxyvitamin D (25OHD) levels with the severity of PD in an Iranian sample. *Methods*. In 109 patients, the severity of PD was evaluated by using Hoehn & Yahr (HR) stages and Unified Parkinson's Disease Rating Stage (UPDRS) Part III compared with 25OHD level in a double-blind and cross-sectional study. *Results*. Mean ± SD levels of 25OHD were 28.5 ± 1.4 and 27.1 ± 1.5, for males and females, respectively. Also, 38.4% of the patients showed deficiency levels of 25OHD (<20 ng/mL), and 72.8% had insufficient levels (<30 ng/mL). High prevalence of 25OHD insufficiency in subjects with early disease was not associated with HR stage and UPDRS scores even after multivariate adjustment for possible confounders including disease duration. *Conclusions*. These findings are consistent with the possibility that vitamin D status does not seem to deteriorate during the early disease stages of PD. Further studies are needed to reveal the natural role and significance of vitamin D insufficiency in PD.

## 1. Introduction

Today, vitamin D is not only considered as a vitamin, but also as a hormone with autocrine and paracrine functions well beyond those of regulating calcium homeostasis and bone health [1]. Optimal balance, muscle strength, and innate immunity require sufficient vitamin D levels, and its deficiency is correlated with increasing risk for various types of cancer, as well as autoimmune and cardiovascular disorders [2–7]. Recently, chronic inadequacy of vitamin D intake has been suggested to play a remarkable role in the pathogenesis or progression of Parkinson's disease (PD) [8]. It seems that the distribution of vitamin D receptors in the substantia nigra is widely known to be affected in PD [9], and the involvement of this vitamin has been revealed in the regulation of tyrosine hydroxylase gene expression and consequently dopamine biosynthesis [10, 11].

Related to the role of vitamin D and PD, there are some cross-sectional studies in Japan indicating that serum levels of 25-hydroxyvitamin D (25OHD) as well as 1, 25-hydroxyvitamin D (1, 25OHD) may have an inverse correlation with the severity of PD [12, 13], and higher circulating 25OHD levels are significantly related to milder form of PD [14]. This observation has been confirmed in a European Caucasian population showing a significant decline in vitamin D levels in patients with PD compared with healthy controls and patients with Alzheimer disease [15]. More recently, in a post hoc analysis of more than 3000 participants in Finland, higher serum vitamin D level was associated with lower risk for PD [8]. But in most of these studies the prevalence of vitamin D deficiency has been shown to be higher in patients with later stage of PD compared to those in early stages; this indicates that having PD and reduced mobility have probably the key role in the relatively high prevalence of vitamin D deficiency. One recent study, however, was an exception showing that not only vitamin D levels in patients with PD did not decrease but also it increased to some extent over the course of followup [10]. Therefore, we designed this study to evaluate vitamin D levels in different stages of PD, to see if there are significant lower levels of vitamin D with disease progression, to elucidate the possible role of vitamin D deficiency and insufficiency in pathogenesis of PD, or as sedentary effect of PD.

Serum 25OHD levels can differ substantially in the same individual depending on the season, latitude, where the person lives, skin color, diet, and other lifestyle factors [16, 17]. On the other hand, there are no reports on PD and vitamin D role focused on the Middle East. In this cross-sectional study, we sought to thoroughly determine associations of circulating 25OHD levels and severity of PD, as well as their interactions in Iranian sample of PD patients.

## 2. Methods

### 2.1. Study Design

This cross-sectional study was carried out at the Alzahra Hospital in Isfahan, Iran, as a double-blind study of 25OHD in patients with PD. 

125 Patients with PD, diagnosed by experienced neurologists, were eligible criteria and asked to participate during September to November 2011. This study was conducted in the outpatient clinics and clinical wards. Patients were excluded if they were already taking vitamin D supplements or 1, 25 vitamin D or were considered to have familial or early onset of PD (<40 years old). Finally, 109 patients had pertinent eligibility for our study.

The diagnosis of PD was based on diagnostic criteria for Parkinson's disease including the presence of resting tremor, bradykinesia, and/or muscle rigidity. Disease duration (months) was defined as the period of time between diagnosis of PD and the clinical assessment for entry into this study. At baseline, we evaluated Hoehn & Yahr (HY) in four stages of 1–1.5, 2–2.5, 3, and 4-5 [18] and also motor part of the Unified Parkinson's Disease Rating Scale III (UPDRS III) [19], by people who were blinded to data regarding 25OHD and serum calcium levels. All patients received standard protocol treatment for PD and provided written informed consent. 

The study protocol was reviewed and approved by the ethics in Research Committee, Isfahan University of Medical Sciences.

### 2.2. Analysis of Laboratory Concentrations

Serum levels of 25OHD (we defined vitamin D insufficiency as a 25OHD concentration of less than 30.0 ng/mL and vitamin D deficiency as a 25OHD concentration of less than 20.0 ng/mL) [10, 20] and parathyroid hormone (PTH) (normal range: 10–65 IU/L) were analyzed by enzyme immunoassay (Biomerica, CA, and IDS, UK). Other laboratory data of peripheral blood including calcium (normal range: 8.2–10.6 mg/dL), phosphorus (normal range: 2.5–4.5 mg/dL), and alkaline phosphatase (ALP) (normal range: 64–306 mg/dL) were performed by spectrophotometric methods (Hitachi 902 autoanalyzer). In each case, serum samples were obtained on the day that HY stage and UPDRS were evaluated, after an overnight fast in acid washed glass tubes. Serum was separated as soon as possible and stored at −20°. 

### 2.3. Statistical Analysis

The effects of 25OHD on the stages of HY were assessed using order logistic regression controlling the variables such as age, gender, disease duration, levels of Ca, phosphorus, PTH, and ALP. Associations between UPDRS Part III and 25OHD were estimated using multiple linear regression models adjustment with age, gender, disease duration, levels of Ca, phosphorus, PTH, and ALP. SPSS statistical software version 16 was used for all statistical calculations. *P* value of less than 0.05 was considered to be significant.

## 3. Results

A total of 109 patients agreed to participate in this study. Mean ± SD values of age and disease duration were 61.4 ± 1.19 years and 57.2 ± 4.94 months, respectively. Severity values of PD measured by UPRDSIII and HY stages with mean ± SD were 22.9 ± 1.81 and 1.8 ± 1.1, respectively.

Most participants were ales 70.6% and 29.4% were females. The mean ± SD values of 25OHD for males and females were 28.5 ± 1.4 and 27.1 ± 1.5, respectively. One-third of the patients (38.4%) showed deficiency levels (<20 ng/mL) and a majority of subjects (72.8%) had insufficient levels (<30 ng/mL) of 25OHD.

**Figure 1:**
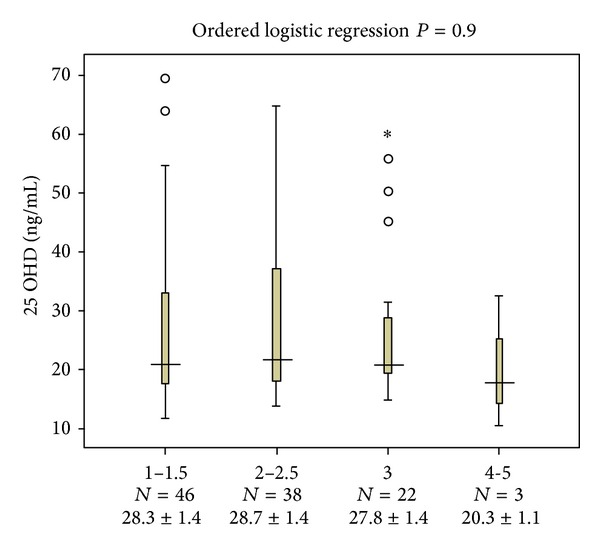
Serum 25OHD levels across Hoehn & Yahr stages. Associations between Hoehn & Yahr stages 1 to 4 and 25OHD levels were assessed using ordered logistic regression after multivariate adjustment for age, gender, disease duration, levels of Ca, Phosphorus, ALP and PTH.

Variants of the study population were divided into 4 groups based on HY staging, as shown in Table 1. Initial analysis showed that patients with higher HY stage had longer duration of disease (*P* = 0.05) and were older (*P* = 0.02). Other characteristics such as gender, Ca, ALP, PTH, and phosphorus did not differ significantly between HY stages (Table 1). There was no association between serum levels of 25OHD with HY stages as shown in Figure 1 (*P* = 0.9). When the severity of PD was evaluated by UPDRS III, there were significant associations between age, duration of disease, and UPDRS (III) (*P* = 0.002 and *P* = 0.003, resp.) (Table 2).

Serum 25OHD levels were not associated with UPDRS III when measured by multiple linear regression methods; however, phosphorus and ALP concentrations were positively related to UPDRS III (*P* = 0.03 and *P* = 0.008, resp.).

## 4. Discussion

In this study, we observed high prevalence of 25OHD insufficiency (72.8%) and deficiency (38.4%). Between the 25OHD levels and HY stages, UPDRS III was not associated even after multivariate adjustment for possible confounders in Isfahan, a sunny city located in the central part of Iran. Positive correlations between UPDRS III and HY scores with age and duration of disease were observed.

Sato et al. reported a negative correlation between 25OHD and not 1, 25 OHD with severity of PD when divided by HY stages 1 to 2 and HY stages 3 to 5 in PD [12]. A higher prevalence of vitamin D deficiency or insufficiency was observed in patients with more advanced disease [12, 13]. Similar results were reported by Suzuki et al., where the severity of PD was evaluated by HY stages 1 to 5. There was a significant trend showing that when HY stage deteriorated, 25OHD levels became lower [14]. It seemed that long-term effects of PD might cause the progression of insufficient vitamin D levels. But these results were not confirmed in our study. The reason for this discrepancy may be contributed to the smaller number of patients with advanced PD in our study—the mean HY stage was 1.8 in our study but 3.3 in Sato et al. study [13] and 2.4 in Suzuki et al. study [14]. At higher HR stages, severe osteopenia is more common, proposing that patients with advanced stages of PD possibly do not go out frequently, and, consequently, may have less sun exposure, which could explain why patients with severe and long-term PD had lower 25OHD concentrations [12, 13].

According to these results, in our study, we observed that the more advanced stage of PD was with lower level of 25OHD (HY stages 1 to 1.5, 25OHD: 28.3 ± 1.4 versus HY stages 4 to 5, 25OHD: 20.3 ± 1.1); but this difference was not significant. Evatt et al. measured 25OHD levels at baseline and at final visits in a longitudinal cohort study and showed that throughout the progression of PD, the level of 25OHD levels did not decline, suggesting that lower circulating 25OHD levels are an accelerator instead of being an outcome of PD [10]. In our study, the observation of mildly significant higher levels of phosphorus and ALP related to higher scores of UPDRS III could be concluded to be the result of mild increased level of hypovitaminosis D in higher stages of PD.

Although both biological and epidemiological data show a contribution of vitamin D deficiency in PD development [21], it is not clear that this correlation is a direct effect or that suffering from a chronic disease has caused reduced mobility and has played a reasonable role in the high prevalence of vitamin D insufficiency [10, 13].

On the other hand, we assessed only 25OHD as an indicator of vitamin D and not 1, 25 OHD. The reason for this choice is related to long half-life of 2 to 3 weeks of 25OHD; it is the best marker of vitamin D status during the last 1 to 2 months, such as a glycosylated hemoglobin level that reflects glucose control [10].

There were several limitations to this study. First, the study design is cross-sectional. Second, the limited information on dietary intake of vitamin D is of potential concern, which could have explained why our patients have a higher concentration of vitamin D compared to previous studies. 

## 5. Conclusion

In conclusion, these findings are consistent with the possibility that vitamin D status does not seem to deteriorate during the early disease stage of PD. Future studies are needed to be conducted on at-risk subjects or presymptomatic to elucidate the potential role of vitamin D insufficiency or deficiency in the pathogenesis or progression of PD.

## Figures and Tables

**Table 1:** Patient characteristics of the study population and association with HY stages.

Characteristics	HY				*P* value	*β* (95% Cl)
	1–1.5 (*n* = 46)	2–2.5 (*n* = 38)	3 (*n* = 22)	4-5 (*n* = 3)		
Age (years) mean ± SD	58.33 ±1.12	63.55 ± 1.10	63.55 ± 1.31	66.25 ± 1.59	0.02	0.03 (0.00, 0.06)
Sex (M/F)	32/20	32/8	17/12	3/1	0.39	0.37 (−0.49, 1.25)
Disease duration (months) mean ± SD	48.00 ± 4.00	63.90 ± 5.50	63.93 ± 4.80	63.25 ± 1.02	0.05	0.00 (0.00, 0.01)
Calcium (mg/dL) mean ± SD	9.30 ± 0.50	9.20 ± 0.44	9.31 ± 0.55	9.40 ± 0.29	0.52	−0.30 (−1.26, 0.64)
Phosphorus (mg/dL) mean ± SD	3.40 ± 0.25	3.34 ± 0.21	3.38 ± 0.18	3.45 ± 0.05	0.27	1.21 (−0.97, 3.40)
ALP (IU/L) mean ± SD	1.76 ± 6.03	1.84 ± 6.18	1.92 ± 7.20	1.63 ± 9.61	0.41	0.00 (−0.00, 0.00)
PTH (ng/mL) mean ± SD	41.80 ± 1.70	46.05 ± 2.16	47.72 ± 1.76	63.66 ± 2.71	0.07	0.01 (−0.00, 0.03)

HY stages—Stage 1: unilateral symptoms only; Stage 1.5: unilateral and axial involvement; Stage 2: bilateral symptoms, no impairment of balance; Stage 2.5: mild bilateral disease with recovery on pull test; Stage 3: balance impairment, mild to moderate disease, physically independent; Stage 4: severe disability, but still able to walk or stand unassisted; Stage 5: needing a wheelchair or bedridden unless assisted.

*P* value was evaluated with single-ordered logistic regression model for HY scale. HY: Hoehn & Yahr; ALP: alkaline phosphatase; PTH: parathyroid hormone; *β*: regression coefficient; CI: confidence interval.

**Table 2:** Summery results of multiple linear regression model to UPDRS III.

Variables	UPDRS III	
	*β* (95% Cl)	*P* value
Age (years)	0.39 (0.14, 0.64)	0.002
Sex (M/F)	−5.05 (−12.1, 1.98)	0.15
Disease duration (months)	0.09 (0.03, 0.15)	0.003
25OHD (ng/mL)	−0.14 (−0.36, 0.06)	0.16
Calcium (mg/dL)	−1.28 (−8.96, 6.39)	0.74
Phosphorus (mg/dL)	18.57 (0.97, 36.18)	0.03
ALP (IU/L)	0.06 (0.01, 0.11)	0.008
PTH (ng/mL)	0.07 (−0.08, 0.23)	0.37

UPDRSIII: unified Parkinson's disease rating stage part III motor; ALP: alkaline phosphates; PTH: parathyroid hormone; 25OHD: 25-hydroxyvitamin D; *β*: regression coefficient; CI: confidence interval.
